# High-Fat Diet–Induced Morphometric Alterations in the Rat Salivary Glands

**DOI:** 10.1055/s-0045-1812060

**Published:** 2025-10-07

**Authors:** Thanit Prasitsak, Komkrith Boonmakum, Kanyanut Tiptirapong, Pokpong Ritkajorn Tungjai, Panuwat Rassaiyakarn, Kroekkiat Chinda, Aubonwan Sitthikhankaew, Siriporn Kreungnium, Thanyaporn Sang-Ngoen

**Affiliations:** 1 Department of Oral Biology, Faculty of Dentistry, Naresuan University, Phitsanulok, Thailand; 2 Doctor of Dental Surgery Program Student, Faculty of Dentistry, Naresuan University, Phitsanulok, Thailand; 3 Department of Physiology, Faculty of Medical Science, Naresuan University, Phitsanulok, Thailand; 4 Integrative Cardiovascular Research Unit, Department of Physiology, Faculty of Medical Science, Naresuan University, Phitsanulok, Thailand

**Keywords:** dyslipidemia, cholesterol, salivary glands, fibrosis, image analysis, simvastatin

## Abstract

**Objectives:**

High-fat diet (HFD) consumption induces metabolic diseases, which lead to salivary gland alteration. However, the alteration in salivary gland remains inconclusive, and the potential protective effect of simvastatin is limited. Therefore, our study aimed to investigate the effect of HFD consumption and the protective effect of simvastatin on submandibular and sublingual glands in rats.

**Materials and Methods:**

Eighteen male Wistar rats were divided into three groups ( *n* = 6 per group): a control group (C) fed a standard diet, a HFD group (H), and a HFD with simvastatin group (S). After 12 weeks, blood was collected for lipid parameter analysis. Submandibular and sublingual glands were stained with hematoxylin and eosin (H&E), Masson's trichrome, periodic acid–Schiff, and alcian blue to evaluate gland architecture, fibrosis, and mucin content. Image analysis was done using imageJ software.

**Statistical Analysis:**

Parametric data were analyzed using one-way ANOVA followed by Tukey's post hoc test. Nonparametric data were analyzed using the Kruskal–Wallis test, followed by Dunn's test. A *p* -value <0.05 was considered statistically significant.

**Results:**

The low-density lipoprotein cholesterol (LDL-C) level significantly increases in the H group compared with the C group ( *p* = 0.004). Acinar cells in both submandibular and sublingual glands were significantly smaller in the H and S groups compared with the C group ( *p* < 0.05). Vacuole-like clear structures were more frequent in the H group. Collagen deposition in the submandibular gland was significantly higher in the H and S groups compared with controls ( *p* = 0.005 and *p* = 0.011, respectively). Slightly altered mucin staining is seen in both glands.

**Conclusion:**

HFD increased LDL-C levels and induced acinar atrophy and fibrosis in the submandibular and sublingual glands. Simvastatin did not protect against salivary gland damage from HFD consumption.

## Introduction

The global rise in obesity and metabolic syndrome has been closely linked to increased consumption of high-fat diet (HFD), which is known to induce systemic metabolic disturbances, including dyslipidemia. These systemic alterations have profound effects on various organs such as the liver, heart, brain, periodontium, etc. 1 2 3 Moreover, the consumption of HFD also affects the salivary glands, which play a crucial role in oral health by maintaining mucosal integrity, initiating digestion, and providing antimicrobial action. 4 5 6

Recent studies have found that HFD can induce significant cellular alterations, which could lead to salivary gland dysfunction. 6 7 8 9 10 11 12 13 For example, HFD disrupts intracellular calcium regulation and enhances reactive oxygen species (ROS) production, leading to mitochondrial dysfunction and apoptosis. 9 12 Some studies investigated the alterations in morphology and functional proteins. Generally, the changes in acini and ductal systems were reported. 6 7 8 10 13 However, a consensus on these changes is lacking in previous studies, and their association with cellular changes remains uncertain.

Statins, such as simvastatin, are widely prescribed lipid-lowering agents. In addition to cholesterol reduction, simvastatin has demonstrated anti-inflammatory and antioxidant properties. 14 15 16 17 One study has reported the protective effects of simvastatin against salivary gland inflammation in arthritis. 18 Another study reported that simvastatin treatment in hyperlipidemic rats resulted in a reduction in lipocyte accumulation in the parotid gland; however, chronic inflammation persisted. 8 The protective effect of simvastatin on the alterations in the submandibular and sublingual glands induced by HFD remains unrevealed.

As the submandibular and sublingual glands play a critical role in maintaining oral health, it is essential to understand the impact of HFD on these glands as well as the potential protective effects of simvastatin. However, current evidence remains inconclusive regarding the effects of HFD, and the protective role of simvastatin remains unrevealed. Therefore, our study aimed to investigate the effect of HFD and the protective effect of simvastatin on histological morphology and mucin production in the submandibular and sublingual glands of rats.

## Materials and Methods

### Animal Ethics

The protocol of animal experiment was approved by the Naresuan University Animal Care and Use Committee under animal ethics no. 6702004. All animals were housed at the Center for Animal Research, Naresuan University, which has been certified by the Association for Assessment and Accreditation of Laboratory Animal Care (AAALAC) International.

### Animal Experiment

The experiment was performed on 7-week-old male Wistar rats ( *Rattus norvegicus* ) obtained from Nomura Siam International Co., Ltd., Bangkok, Thailand. Constant environmental conditions, i.e., 12-hour light/dark cycle, stable temperature of 22 ± 1°C, and relative humidity of 55 ± 10%, were maintained throughout the entire study. After 1 week of an acclimation period, the rats were randomly divided into three equally numbered groups of six animals: (1) control (C) group, (2) HFD (H) group, and (3) HFD with simvastatin (S) group.

Each group received a distinct diet for 12 weeks. The C group was fed a standard diet (SmartHeart Hamster Food Complete & Balance, Perfect Companion Group Co., Ltd., Formula Code 082), consisting of 4.5% fat, 24.2% protein, and 44.8% carbohydrates. Rats in the H and S groups were fed with HFD (27.42% fat, 11.85% protein, 41.94% carbohydrates). The S group additionally received 40 mg/kg/day of simvastatin, administered alongside the HFD. Food consumption and body weight were monitored weekly.

After the 12-week experimental period, all rats underwent a 12-hour fasting period. Anesthesia was induced via intraperitoneal injection of sodium thiopental (50 mg/kg body weight). Blood samples were subsequently collected from the inferior vena cava. Both submandibular and sublingual salivary glands were then harvested and immediately immersed in 4% paraformaldehyde prepared in phosphate-buffered saline at 4°C for 2 days.

### Blood Cholesterol Analysis

Serum samples were analyzed for total cholesterol (TC), low-density lipoprotein cholesterol (LDL-C), high-density lipoprotein cholesterol (HDL-C), and triglyceride (TG) levels using an automated analyzer (Cobas c 311 analyzer, Roche).

### Histological Section and Staining

The submandibular and sublingual glands were subsequently processed and embedded in paraffin after fixation, then sectioned at a thickness of 4 µm, and stained with hematoxylin and eosin (H&E) to assess general tissue architecture. Masson's trichrome staining was performed to detect collagen deposition (fibrosis), periodic acid–Schiff (PAS) staining was used to visualize neutral mucins, and alcian blue staining was applied to identify acidic mucins.

### Histological Analysis

Stained tissue sections were scanned using a slide scanner (ZEISS Axio Scan.Z1) equipped with an LED light source (ZEISS Colibri 7) and image acquisition software (ZEN Blue 2.3). Quantitative histological analysis was conducted using ImageJ software.

Both submandibular and sublingual glands were analyzed for acinar cell size, striated duct size, and the percentage area occupied by collagen. The process of image analysis was blinded by assigning number codes for the images. The investigator performing the analysis was unaware of the experimental groups. At the end of the experiment, the number codes were revealed to collect the data and perform statistical analysis.

For acinar cell size, five nonoverlapping images at 200× magnification were selected per sample. These images were taken from five distinct regions: two from the outer edges of the gland, two from the central area, and one from the region adjacent to the neighboring gland. In each image, the total acinar area was measured in μm ^2^ and divided by the number of visible nuclei to calculate the average acinar cell size. The values from all five images were then averaged to obtain a representative value for each animal, which was subsequently used to calculate group means.

For striated ducts, 10 nonoverlapping areas containing striated ducts at 200× magnification were selected. The duct area was measured in μm ^2^ and based on clearly defined and traceable duct boundaries. Approximately 20 to 30 ducts were yielded from each rat.

The percentage area of collagen was calculated from Masson's trichrome-stained sections by selecting blue-stained regions and dividing by the total gland area, then multiplying by 100. The data were shown as a percentage of the total gland area.

### Statistical Analysis

The serum lipid parameters, the average acinar cell size of the submandibular gland, and the mean percentage areas of collagen are presented as means ± standard deviation (SD). These data were analyzed using one-way analysis of variance (ANOVA) followed by Tukey's post-hoc test. The average acinar cell size of the sublingual gland was nonparametric data, presented as median and quartiles 1 and 3. These data were analyzed using the Kruskal–Wallis test followed by Dunn's test. The size of striated ducts is presented as median, minimum, and maximum. All statistical analyses were performed using JASP software (Version 0.17.2; JASP Team, 2024), 19 and a *p* -value of < 0.05 was considered statistically significant.

## Results

### Blood Lipid Profiles

LDL-C levels differed significantly across all groups ( *p* = 0.006), whereas TC, HDL-C, and TG levels showed no statistically significant differences ( *p* = 0.129, 0.065, and 0.157, respectively). HFD consumption resulted in increased levels of all lipid parameters in the H group compared with the C group, but only the increase in LDL-C was statistically significant ( *p* = 0.004). Simvastatin treatment (S group) reduced all serum lipid levels compared with the H group; however, these reductions were not statistically significant. Additionally, TC, HDL-C, and TG levels in the S group were lower than those in the C group, while the LDL-C level was higher. Nevertheless, none of these differences were statistically significant ( Table 1 ; Supplementary Tables S1 and S2 , available in the online version only).

**Table 1:** Blood lipid profiles of rats in the standard diet (C), high-fat diet (H), and high-fat diet with simvastatin (S) groups. Values are presented as mean ± standard deviation (SD) for total cholesterol (TC, mg/dL), high-density lipoprotein cholesterol (HDL-C, mg/dL), low-density lipoprotein cholesterol (LDL-C, mg/dL), and triglyceride (TG, mg/dL)

Blood profile	Control	High-fat diet	High-fat diet with simvastatin
***N***	6	6	6
**TC (mg/dL)**	57.8 ± 16.9	86.2 ± 30.2	55.5 ± 8.67
**HDL-C (mg/dL)**	35.7 ± 11.1	39.3 ± 14.2	23.7 ± 6.44
**LDL-C (mg/dL)**	9.50 ± 3.78 ^a^	37.0 ± 19.8 ^b^	20.2 ± 6.24 ^a,b^
**TG (mg/dL)**	28.8 ± 15.3	33.0 ± 12.7	19.3 ± 5.09

Note: Groups sharing the same letter (a, b) are statistically **not different** , while those with different letters are **significantly different** .

### Histological Analysis

Submandibular and sublingual glands were observed as follows:

#### Acinar Units

**Fig. 1:**
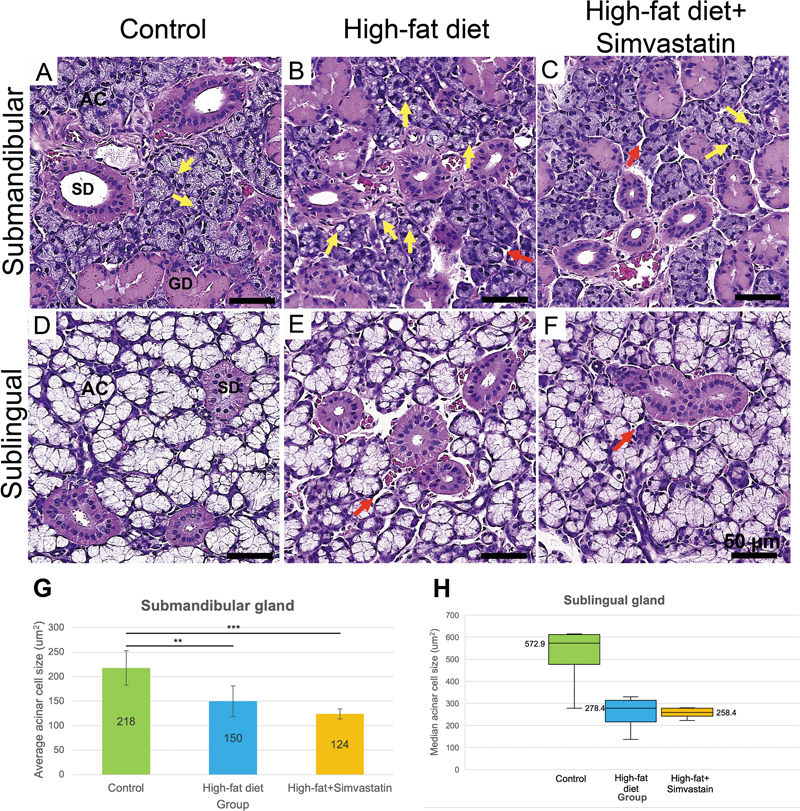
High-fat diet consumption reduced acinar cell size in both submandibular and sublingual glands. Hematoxylin and eosin staining of the submandibular ( **A–C** ) and sublingual ( **D–F** ) glands. Intercellular spaces (red arrow) were evident in the high-fat diet (B, E) and the high-fat diet with simvastatin (C, F) groups compared with control (A, D), suggesting reduced size of acinar cells in both glands. In addition, numerous cytoplasmic vacuoles (yellow arrow) were observed in the acinar cells of all groups, but prominently seen in the high-fat diet group (A–C). Data of the submandibular gland shown as mean ± SD ( **G** ). Data of the sublingual gland shown as median and quartile ( **H** ). Scale bars: 50 μm. ** *p* < 0.01, *** *p* < 0.001. AC, acini; GD, granular duct; SD, striated duct; SD, standard deviation.

In the submandibular gland, all groups displayed similar morphology. Normal acinar cells with purple-red cytoplasm and basally located nuclei were observed ( Fig. 1A–C ). The acinar units in the C group appeared larger in size and densely packed ( Fig. 1A ) compared with acinar units in H and S groups, which exhibit smaller acinar units with visible spaces between some units ( Fig. 1B, C , red arrow). In addition, the H group represented a higher number of clear round structures inside the cell ( Fig. 1A–C , yellow arrows) compared with the C and S groups. These structures resembled vacuoles or lipid droplets. However, oil red O staining, which indicates the presence of lipid droplets, showed similar staining patterns among the groups (data not shown), suggesting an increase in non-lipid vacuoles in theses group. Next, the average size of acinar cells was measured (shown in Fig. 1G ; Supplementary Tables S3 and S4 , available in the online version only) and found to be significantly smaller in both the H and S groups compared with the C group ( *p* = 0.002 and *p* < 0.001, respectively). No significant difference in acinar size was observed between the H and S groups ( *p* = 0.274).

Similarly, acinar units in the sublingual glands of all groups exhibited comparable morphology ( Fig. 1D–F ). Smaller acinar units with visible spaces between some of the units in the H and S groups ( Fig. 1E, F , red arrow) were observed. In contrast, more densely packed acinar units were revealed in the C group ( Fig. 1D ). However, vacuoles in the acinar cells were not detected. Consistent with the submandibular gland, a significantly smaller acinar cell size was found in both the H and S groups compared with the C group ( *p* = 0.017, *p* = 0.005, respectively; Fig. 1H , Supplementary Tables S3 and S4 , available in the online version only). However, no significant difference was exhibited between the H and S groups ( *p* = 0.665).

#### Ducts

Both the submandibular and sublingual glands contained excretory ducts, striated ducts, and intercalated ducts, each characterized by distinct epithelial morphologies. In the submandibular gland, prominent convoluted (granular) ducts with red-stained cytoplasm were also observed. All ductal cells exhibited well-defined cell borders, and no obvious morphological changes were noticed across the groups.

Striated ducts were lined by cuboidal to columnar cells with centrally located dark nuclei and red-stained cytoplasm (SD; Fig. 1A–F ). The size of the striated ducts in both glands varied across individual rats; the sizes were found to be similar between the groups. The median, minimum, and maximum size of striated ducts in both glands are presented in Supplementary Fig. S1 , available in the online version only. No distinct morphological changes were observed across the groups.

#### Collagen Deposition

**Fig. 2:**
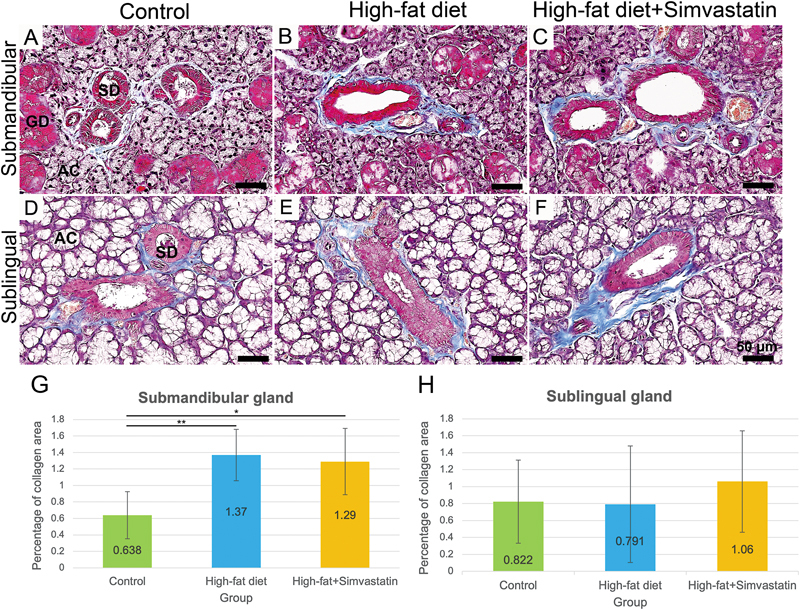
Collagen deposition surrounding salivary ducts increased in response to high-fat diet consumption. Masson's trichrome staining of the submandibular ( **A–C** ) and sublingual ( **D–F** ) glands presented collagen accumulation around salivary ducts. Collagen appeared as light blue in the control group (A, D) and showed increased intensity in the high-fat diet (B, E) and high-fat diet with simvastatin (C, F) groups. Data shown as mean ± SD in submandibular ( **G** ) and sublingual ( **H** ) glands. Scale bars: 50 μm. * *p* < 0.05, ** *p* < 0.01. AC, acini; GD, granular duct; SD, striated duct; SD, standard deviation.

Masson's trichrome staining revealed collagen as blue-stained areas, as shown in Fig. 2(A–F) . In both salivary glands, collagen accumulation was predominantly observed in the periductal area, particularly around large ducts. Light blue staining was observed in the C group ( Fig. 2A, D ), particularly in the submandibular gland, while more intense blue coloration was evident in the H and S groups ( Fig. 2B, E and 2C, F , respectively). Quantitative analysis ( Fig. 2G, H ) demonstrated a statistically significant increase in collagen deposition in the submandibular gland among the three groups ( *p* = 0.003). In the submandibular gland ( Fig. 2G ), statistically significant differences were found in both the H and S groups compared with the C group ( *p* = 0.005 and *p* = 0.011, respectively), with no significant difference between the H and S groups ( *p* = 0.914). A similar pattern of collagen deposition was observed in the sublingual gland ( Fig. 2H ); however, the differences among groups were not statistically significant ( *p* = 0.699).

#### Mucin

**Fig. 3:**
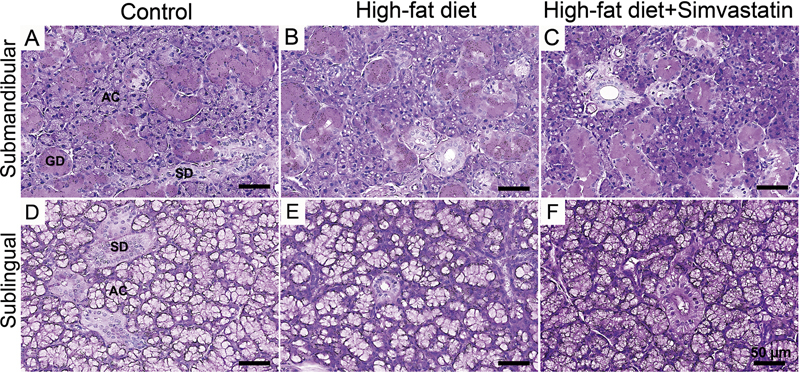
Periodic acid–Schiff (PAS) staining revealed neutral mucin in the submandibular and sublingual glands. Magenta staining indicates the presence of neutral mucin within acinar cells ( **A–F** ). The prominent staining was observed in serous acini of the submandibular gland (A–C). Sublingual glands show increased intensity of the staining in the surrounding areas of H and S groups (D–F). Scale bars: 50 μm. AC, acini; GD, granular duct; SD, striated duct.

The magenta coloration in PAS staining ( Fig. 3A–F ), indicating the presence of neutral mucin, was prominently observed in the acini of the submandibular gland, and increased intensity of the staining was observed in the H and S groups ( Fig. 3A–C ). The staining was also found in the periacinar area of the sublingual gland, particularly H and S groups ( Fig. 3D–F ).

**Fig. 4:**
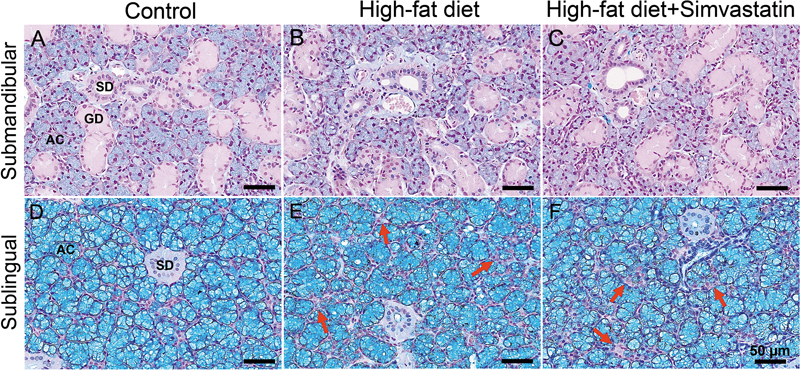
Alcian blue staining revealed acidic mucin in submandibular and sublingual glands. Blue staining denotes acidic mucin within acinar cells of both glands ( **A–F** ). The staining was prominently observed in the sublingual gland (D–F). Additionally, thicker pale pink structures surrounding acini (red arrow) were observed in the sublingual gland of H and S groups. Scale bars: 50 μm (A–F). AC, acini; GD, granular duct; SD, striated duct.

Alcian blue staining ( Fig. 4A–F ), indicating the presence of acidic mucin, was prominently observed in the acinar cells of the sublingual gland ( Fig. 4D–F ). Additionally, thicker pale pink structures with dark-staining nuclei surrounding the mucous acini were observed in the H and S groups ( Fig. 4E, F , red arrow).

## Discussion

HFD consumption is known to elevate serum lipid levels—including TC, LDL-C, and TGs, which contribute to systemic metabolic disturbances that affect multiple organ systems. Secretory glands, such as the liver, pancreas, and lacrimal gland, are particularly vulnerable to the changes, as they play vital roles in maintaining homeostasis. 20 21 22 Among these, the salivary glands are essential for preserving oral health and homeostasis; the impact of HFD on their structure and function remains inconclusive—particularly in relation to the submandibular and sublingual glands.

Our findings demonstrate that HFD consumption induces significant morphological alterations in both glands, including a marked reduction in acinar cell size and increased collagen deposition, and minimal mucin alteration. These structural changes were associated with elevated LDL-C levels. Simvastatin treatment did not significantly prevent these alterations.

Animal models, particularly rats, are widely used for organ changes associated with metabolic diseases. 23 HFD feeding in rats is a well-established method for inducing dyslipidemia and other metabolic disturbances. 23 24 A previous study has reported that rat diets containing 20 to 60% fat can trigger various metabolic abnormalities. 23 However, the specific formulation of the diet plays a crucial role, as different fat compositions have been shown to produce varying blood lipid profiles. 13 24 25 26 In our study, we used a diet containing 24.2% fat, which is on the lower end compared with other experimental HFD models. 12 24 26 27 Nevertheless, our diet formula was sufficient to increase LDL-C and TC levels in the H group, indicating that metabolic changes were successfully induced in our rat model. Administration of simvastatin at a moderate dose (40 mg/kg) resulted in a reduction of LDL-C levels, consistent with previous findings showing that simvastatin can lower LDL-C by approximately 30 to 49% through inhibition of HMG-CoA reductase activity. 28 These results confirm the effectiveness of simvastatin treatment in our study.

To date, only a few studies have examined the morphological effects of HFD on major salivary glands, and the alteration remains inconclusive. 6 7 8 10 11 13 The enlarged acinar cells were reported in the parotid and submandibular glands, while shrinkage in acini was reported in the sublingual gland. 8 10 13 However, the cytoplasmic vacuolization was commonly found in all major salivary glands. 6 7 10 In addition, ductal alterations, for example, dilated ducts, a decreased number of ducts, and changes in epithelial lining in the parotid and submandibular glands were observed in some studies. 6 7 8 In our study, we found acinar shrinkage, but ductal changes were inconspicuous.

The discrepancies among the previous studies and our findings gave us two observations. First, the duration of HFD feeding might affect the characteristics of the damage. In the submandibular gland, Kandeel and Elwan 10 reported the enlargement of acinar cells in rats fed a HFD for 6 weeks. In contrast, our 12-week HFD feeding model showed acinar cell shrinkage. When a similar feeding duration (12 weeks) was applied in our model and Hamada et al, 13 acinar cell shrinkage was reported in the sublingual gland. Second, different types of salivary glands may respond differently to HFD. In a 12-week HFD feeding model, the parotid gland was enlarged, 8 whereas the submandibular and sublingual glands were reduced in size, as shown in our study. Most previous studies focused on alteration in a single gland, suggesting that future research should investigate multiple glands to obtain more consistent results.

Increased collagen deposition—indicative of fibrosis—is a common finding and is consistently reported across previous studies, and our results align with this observation. 6 8 Additionally, our findings from PAS and alcian blue staining revealed a thicker periacinar area in the sublingual glands of the H and S groups, suggesting potential alterations in the surrounding components, such as the basement membrane. 29 The accumulation of extracellular matrix components, including collagen, is considered a hallmark of tissue damage and a precursor to fibrosis. 30 31

Our study observed a slightly increased PAS staining intensity in the acinar cells of the H group, a finding consistent with a previous report that also noted increased alcian blue intensity in the sublingual gland. 13 Only a few studies have reported on mucin in the salivary gland under HFD conditions. However, a report of increased mucin in the gallbladder suggests a link to inflammation and fibrosis. 32 Thus, the mucin alteration in our study possibly relates to inflammation and the protective mechanism of the organ. Nevertheless, no recent evidence has reported these changes.

Taken together, HFD consumption leads to alterations in glandular and surrounding structures. The alterations provide evidence of early tissue injury, which may progress toward apoptotic activity and, ultimately, fibrosis. 30 31 These collective cellular alterations may impair salivary gland function, consequently, a decrease in salivary flow, which is a clinical manifestation of xerostomia. 5 33

Simvastatin, widely used for cholesterol lowering, has been reported to have anti-inflammatory and antifibrotic properties in the liver and parotid gland. 18 34 Daskala and Tesseromatis reported that simvastatin significantly reduced blood lipid parameters, but insignificantly mitigated ductal and acinar cell alterations in the parotid glands of HFD-fed rats. 8 Similarly, simvastatin reduced serum cholesterol levels, but did not significantly protect against alterations in glandular and gland-surrounding structures in our study. We suggest that simvastatin is ineffective in glandular protection from HFD consumption in our model.

Our study is limited by the use of histological analysis, which represents morphology and structures. However, morphology might not disclose a distinct alteration in the early stage, but the functions of the organ have already changed. 35 To gain a more comprehensive understanding of the cellular and molecular changes induced by HFD consumption, future studies should incorporate molecular techniques—such as immunohistochemistry, gene expression profiling, or protein assays—to validate and extend our histological findings. Combining morphological and molecular approaches would provide stronger evidence for the mechanisms underlying salivary gland alterations in metabolic disorders.

## Conclusion

HFD consumption led to elevated LDL-C levels and induced acinar cell shrinkage and increased collagen deposition. The alteration in mucin requires further molecular studies to assess glandular function. Simvastatin did not prevent structural changes and fibrosis in the glands. These findings highlight the vulnerability of salivary glands to HFD, raising clinical concern for oral health risks and the imperative protective strategies.
