# Supplementary material for: High-Fat Diet–Induced Morphometric Alterations in the Rat Salivary Glands
Source: Eur J Dent. 2025 Oct 7;20(3):996–1003. doi: 10.1055/s-0045-1812060 (PMC13337257; doi:10.1055/s-0045-1812060)
Supplement: Supplementary file 1 — Supplementary Material [file 10-1055-s-0045-1812060-s2564331.pdf]

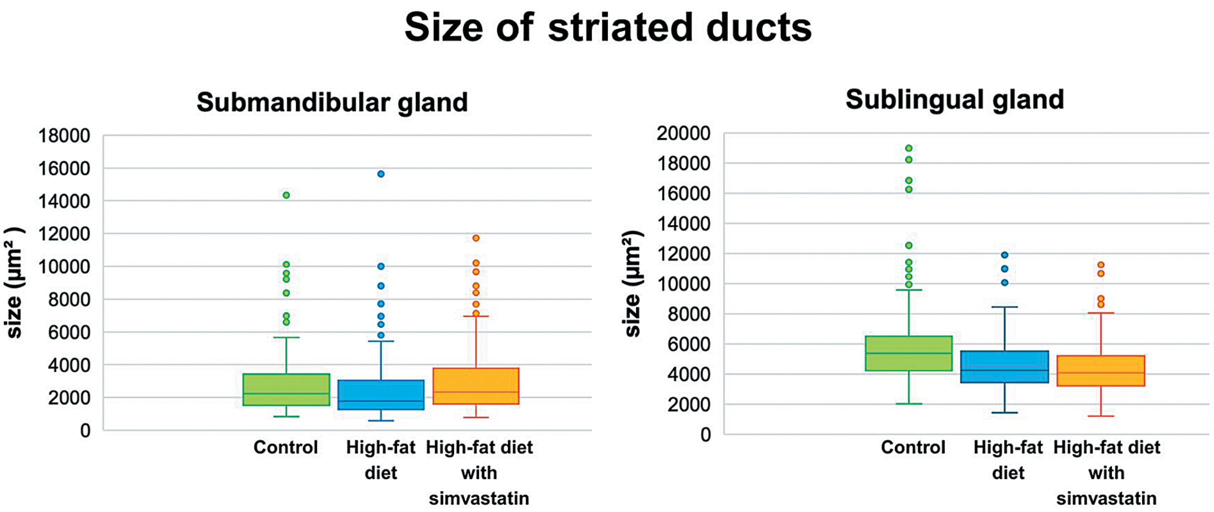

Supplementary Fig. S1 The size of straited ducts in the submandibular and sublingual glands. A plot graph showing the minimum, median, quartile 1, quartile 3, and the maximum size of straited ducts of three groups in the submandibular (left) and sublingual (right) glands.

Supplementary Table S1 Comparison of blood lipid profiles in rats from the control, high-fat diet, and high-fat diet with simvastatin groups. Values are expressed as mean ± standard deviation (SD) for total cholesterol (TC, mg/dL), high-density lipoprotein cholesterol (HDL-C, mg/dL), low-density lipoprotein cholesterol (LDL-C, mg/dL), and triglycerides (TG, mg/dL). Statistical analysis was performed using one-way ANOVA. *p*-Value and Eta-squared (η^2) were reported

Blood lipid profile	Control	High-fat diet	High-fat diet with simvastatin
N	6	6	6
TC (mg/dL)			
Mean ± SD	57.8 ± 16.9	86.2 ± 30.2	55.5 ± 8.67
p-value (one-way ANOVA)	0.129		
η^2	0.355		
HDL-C (mg/dL)			
Mean ± SD	35.7 ± 11.1	39.3 ± 14.2	23.7 ± 6.44
p-Value (one-way ANOVA)	0.065		
η^2	0.305		
LDL-C (mg/dL)			
Mean ± SD	9.50 ± 3.78	37.0 ± 19.8	20.2 ± 6.24
p-Value (one-way ANOVA)	0.006**		
η^2	0.510		
TG (mg/dL)			
Mean ± SD	28.8 ± 15.3	33.0 ± 12.7	19.3 ± 5.09
p-Value (one-way ANOVA)	0.157		
η^2	0.218		

**$p < 0.01$.

 © 2025. The Author(s).

Supplementary Table S2 Post-hoc analysis of blood lipid profiles in rats from the normal diet, high-fat diet, and high-fat diet with simvastatin groups. *p*-Values and 95% confidence intervals are reported for total cholesterol (TC, mg/dL), high-density lipoprotein cholesterol (HDL-C, mg/dL), low-density lipoprotein cholesterol (LDL-C, mg/dL), and triglycerides (TG, mg/dL). *p*-Values and Cohen's d are reported

Blood profile	TC (mg/dL)		HDL-C (mg/dL)		LDL-C (mg/dL)		TG (mg/dL)	
	p-Value	Cohen's d	*p*-Value	Cohen's d	*p*-Value	Cohen's d	*p*-Value	Cohen's d
**High-fat diet vs. normal diet**	0.075	1.376	0.836	0.332	0.004*	2.260	0.817	0.352
**High-fat diet with simvastatin vs. normal diet**	0.979	−0.113	0.179	−1.085	0.311	0.877	0.371	−0.802
**High-fat diet vs. high-fat diet with simvastatin**	0.052	1.489	0.065	1.417	0.073	1.384	0.147	1.153

*$p < 0.05$.

Supplementary Table S3 Comparison of average acinar cell size and percentage of collagen area in the submandibular and sublingual glands of rats in the normal diet, high-fat diet, and high-fat diet with simvastatin groups. For parametric data, mean ± standard deviation (SD) is presented, and one-way ANOVA was applied for statistical analysis. For nonparametric data, median and quartiles 1 and 3 are presented, and the Kruskal–Wallis test was applied. *p*-Values, Eta-squared (η^2), and Epsilon-squared (ε^2) were reported

Parameters	Control	High-fat diet	High-fat diet with simvastatin
**Submandibular gland**			
**Acinar cell size (μm²)**			
**Mean ± SD**	218.5 ± 35.16	149.8 ± 31.46	124.0 ± 10.22
p-Value (one-way ANOVA)	<0.001***		
η^2	0.711		
**Collagen area (%)**			
**Mean ± SD**	0.638 ± 0.286	1.371 ± 0.311	1.293 ± 0.403
p-Value (one-way ANOVA)	0.003**		
η^2	0.533		
**Sublingual gland**			
**Acinar cell size (μm²)**			
**Median (Q1, Q3)**	572.9 (549.9–601.4)	278.4 (245.6–306.1)	258.4 (250.5–273.2)
***p*-value** (Kruskal–Wallis test)	0.010**		
ε^2	0.539		
**Collagen area (%)**			
**Mean ± SD**	0.822 ± 0.490	0.791 ± 0.688	1.061 ± 0.599
***p*-Value** (one-way ANOVA)	0.699		
η^2	0.047		

**$p < 0.01$, ***$p < 0.001$.

Supplementary Table S4 Pairwise comparison of average acinar cell size and percentage of collagen area in submandibular and sublingual glands of rats in the normal diet, high-fat diet, and high-fat diet with simvastatin groups. All parameters except the acinar cell size of the sublingual gland were analyzed by using post-hoc test. *p*-Values and Cohen's *d* are reported. The acinar cell size of the sublingual gland was analyzed by using Dunn's post-hoc test. *p*-Values and rank-based correlation (*r*) are reported

Parameters	Submandibular gland				Sublingual gland			
	Acinar cell size (μm²)		Collagen area (%)		Acinar cell size (μm²)		Collagen area (%)	
	p-Value	Cohen's *d*	*p*-Value	Cohen's *d*	*p*-Value	r	*p*-Value	Cohen's *d*
**High-fat diet vs. normal diet**	0.002**	−2.463	0.005**	2.175	0.017*	0.688	0.996	−0.052
**High-fat diet with simvastatin vs. normal diet**	<0.001***	−3.389	0.011*	1.941	0.005**	0.813	0.772	−0.399
**High-fat diet vs. high-fat diet with simvastatin**	0.274	0.926	0.914	0.233	0.665	0.125	0.719	−0.452

*$p < 0.05$, **$p < 0.01$, ***$p < 0.001$.

 © 2025. The Author(s).
